# Efficacy and safety of endovascular thrombectomy in mild ischemic stroke: results from a retrospective study and meta-analysis of previous trials

**DOI:** 10.1186/s12883-019-1372-9

**Published:** 2019-07-05

**Authors:** Xian-Jin Shang, Zhong-Hua Shi, Cai-Feng He, Shuai Zhang, Yong-Jie Bai, Yong-Tao Guo, Bo Sun, Shun Li, Huai-Ming Wang, Zhi-Ming Zhou, Wen-Jie Zi, Xin-Feng Liu

**Affiliations:** 1 Department of Neurology, Jinling Hospital, Jinling Clinical College of Nanjing Medical University, Nanjing, 210002 Jiangsu China; 2 Department of Neurology, Yijishan Hospital of Wannan Medical College, Wuhu, 241001 Anhui China; 3 Department of Neurosurgery, The 101st Hospital of the People’s Liberation Army, Wuxi, 214000 Jiangsu China; 4 Department of Dermatology, Yijishan Hospital of Wannan Medical College, Wuhu, 241001 Anhui China; 5 Department of Neurology, The affiliated Hospital of Yangzhou University, Yangzhou, 225001 Jiangsu China; 6 Department of Neurology, First Affiliated Hospital, and College of Clinical Medicine of Henan University of Science and Technology, Luoyang, 471003 China; 7 Department of Neurology, The Affiliated Huai’an NO.1 People’s Hospital, Nanjing Medical University, Huai’an, 223300 Jiangsu China; 8 Department of Neurology, Jinling Hospital, Southern Medical University, Nanjing, 210002 Jiangsu China; 9 Department of Neurology, Jinling Hospital, Medical School of Nanjing University, Nanjing, 210002 Jiangsu China

**Keywords:** Thrombectomy, Stroke, Endovascular, Outcome

## Abstract

**Background:**

Mechanical thrombectomy has been proven as a standard care for moderate to severe ischemic stroke with anterior large vessel occlusion (LVO); however, whether it is equally effective in mild ischemic stroke (MIS) is controversial.

**Methods:**

In this retrospective study, a total of 177 Chinese patients presenting with MIS (NIHSS ≤8) and LVO between January 2014 and September 2017 from seven comprehensive stroke centers were identified. Odds of good outcome with endovascular thrombectomy versus medical treatment were obtained by logistic regression analysis and propensity-score matching method, and a meta-analysis pooled results from six studies (*n* = 733).

**Results:**

Good outcome (mRS: 0–1) was 58.2% (46/79) in the thrombectomy and 46.9% (46/98) in the medical group, which showed no statistical significance before adjustment (*P* = 0.13; OR = 1.57, 95% CI: 0.86 to 2.86). The adjusted ORs of thrombectomy versus medical group were 3.23 (95% CI, 1.35 to 7.73; *P* = 0.008) by multivariable logistic analysis, 2.78 (1.12 to 6.89; *P* = 0.02) by propensity score matching analysis, and 3.20 (1.22 to 8.37; *P* = 0.01) by propensity score matching analysis with additional adjustments, respectively. Thrombectomy treatment did not result in excessive mortality or symptomatic intracranial hemorrhage after adjustments. The meta-analysis did not confirm the associations between good outcome and endovascular treatment.

**Conclusions:**

The current study indicates that endovascular thrombectomy is associated with good functional outcome in MIS patients with LVO, and without additional risk of symptomatic intracranial hemorrhage and mortality. Although the meta-analysis failed to demonstrate its superiority compared to medical treatment, randomized clinical trials are needed.

**Electronic supplementary material:**

The online version of this article (10.1186/s12883-019-1372-9) contains supplementary material, which is available to authorized users.

## Background

Since the publication of six randomized control trials, mechanical endovascular thrombectomy (MET) has become a standard therapy for patients with moderate to severe ischemic stroke and large vessel occlusion (LVO) in the proximal anterior circulation [1–6]. Mild ischemic stroke (MIS) accounts for > 15% of acute ischemic stroke, and patients with LVO also have a risk of severe deterioration if there is no reperfusion [7–9]. Endovascular therapy was reported to rapidly and effectively recanalize the occluded vessel, but also increased the risk of intracranial hemorrhage associated with no improvement of clinical functional outcome [10, 11]. Conversely, endovascular treatment was reported to improve the clinical symptoms of patients with MIS during hospitalization, and also improve long-term prognosis [12]. Therefore, the efficacy and safety of MET for MIS remains controversial.

In this study, we retrospectively analyzed the data of MIS patients treated with MET or medical therapy from our center, and combined the previously published data through a meta-analysis to obtain more reliable conclusions.

## Methods

### Patient selection

We conducted a retrospective study involving patients with acute ischemic stroke who were consecutively admitted to 7 comprehensive stroke centers from China (Jinling Hospital, Yijishan Hospital, Fuzhou General Hospital of Nanjing Military Region, Affiliated Hospital of Yangzhou University, Daping Hospital, Hubei Zhongshan Hospital, and No.123 Hospital of the People’s Liberation Army) between January 2014 and August 2017, and all these patients initially presented with mild neurological deficit during 24 h, which defined as NIHSS ≤8 (because the cut-off of 8 always recognized as probably having LVO), and was diagnosed as having LVO, including ICA, M1 or M2 segment of MCA, and ACA on CTA or MRA. The specific exclusion criteria were: (i) admission age < 18 years, (ii) patients who had a prior modified Rankin Score (mRS) ≥2, and (iii) arteriovenous malformation and arterial aneurysm determined by CTA/MRA.

All eligible subjects were divided into the MET group or the medical group, according to whether MET was performed. The MET group included the initial MET and rescue MET [13]. The local Ethics Committees of each center approved the use of patients’ data for this study, and written consents of the patients were waived due to its retrospective nature.

### Data collection

We retrieved demographic, clinical, and neuroimaging data from all eligible subjects, including age, sex, stroke risk factors (atrial fibrillation, hypertension, dyslipidemia, diabetes and smoking), admission systolic blood pressure, admission NIHSS scores, time of onset to imaging, use of intravenous thrombolysis, and the ASPECT score and collateral circulation assessment based on admission imaging data. Good collateral circulation indicated rapid or complete collateral collateral flow into the ischemic area. Stroke etiology was defined according to the Trial of Org 10,172 in Acute Stroke Treatment classification, and was grouped into large atherosclerosis, cardioembolism, and others/undetermined [14]. Stroke occlusion location was divided into ICA, MCA (M1 or M2 segments), and tandem occlusion corresponding to the artery occlusion occurring in more than two different parts of a continuous vessel.

All radiological data and clinical medical records of the subjects were sent to the core laboratory in our hospital, and were reviewed in a blinded fashion by two neurologists (Y-T Guo and S Zhang) with advice of a third experienced neurologist (W-J Zi) in cases of disagreement.

### Clinical outcomes

The patients’ functional outcomes at 3 months were measured by the mRS, ranging from 0 to 6 (higher scores indicate more severe disability), which was collected by telephone follow-up or outpatient visit in each center by staff with a structured interview [15]. Symptomatic intracranial hemorrhage (sICH) was considered as any hemorrhage combined with an increase of ≥4 points in total NIHSS score, or ≥ 2 points in one NIHSS category, according to the Heidelberg classification scheme [16]. The primary outcome of this study was good outcome defined as an mRS 0–1, while secondary outcomes included favorable outcome as mRS 0–2, mortality at 3 months and sICH at 48 h.

### Meta-analysis

A pooled analysis of studies comparing endovascular with medical treatment, either with or without intravenous tissue plasminogen activator, in patients with mild stroke and LVO was conducted. We retrieved the relevant studies from four electronic databases including Pubmed, Embase, Cochrane Database of Systematic Reviews and Cochrane Central Register of Controlled Trials from database inception to May 2018, and used the appropriate free text and Mesh terms to identify them: “mild stroke”, “minor stroke”, “minimal stroke”, “NIHSS≤8”, and “recanalization”, “endovascular”, “thrombectomy”, “stent-retriever”, “thrombolysis”, and “reperfusion”. Additional records were identified through reference lists of eligible studies.

All the retrieved studies were screened by two independent reviewers (X-J Shang and C-F He) according to the following criteria: (i) MIS patients with LVO (ICA, MCA M1 and M2, ACA, posterior circulation), defined as baseline NIHSS ≤8 at onset, (ii) all studies selected included endovascular or thrombectomy treatment, and case reports, abstracts, editorials and expert opinions were excluded, and (iii) all studies selected should be human trials and in English, and if more than one trials came from the same center with the same dataset, only the most complete dataset published was used for final analysis. The third reviewer (W-J Zi) assessed the quality of observational trials and cohort studies using the Newcastle-Ottawa scale, and coordinated and resolved the discrepancies between reviewers.

### Statistical analysis

Qualitative variables were shown as number and percentage, and for quantitative variables, normally distributed data were presented as mean and standard deviation, while non-normally distributed data were presented as median and interquartile range. The difference in baseline characteristics between MET group and medical group was assessed using calculating the absolute standardized difference (ASD), and ASD > 0.10 was recognized as significant difference.

We adopted the following strategies to compare clinical outcomes between the two groups: (i) Multivariable logistic regression was performed using variables with ASD > 0.10 on comparisons of baseline characteristics between the two groups; (ii) Propensity score (PS) matching method was used to reduce the effects of potential confounding factors on between-group comparisons, and calculated the odds ratio (OR) for the MET versus medical therapy as the treatment effect size. (iii) If the baseline differences still existed after the PS matching, we included variable factors with ASD > 0.10 into the logistic regression equation to further calculate the treatment effect size.

In the meta-analysis, the possible clinical or methodological variation had been taken into account, so we adopted a randomized model. We used χ^2^ tests to assess heterogeneity between trials and I^2^ statistic to estimate the percentage of total variation across studies, with values beyond 50% regarded as substantial heterogeneity.

Significance was set at *P* < 0.05 using two-sided tests. All statistical analyses were performed with Statistical Software (IBM SPSS Statistics 22; IBM-Armonk, New York, USA), and Review Manager Version 5.3.4 (Cochrane Collaboration, Software Update, Oxford, United Kingdom).

## Results

### Baseline characteristics

A total of 79 patients treated with MET and 98 patients having medical treatment were identified in this study. The Table 1 shows the baseline characteristics of two groups before and after PS-matching. Most of the variables had substantial differences (ASD > 0.10), except for the hypertension history and ASPECTS before matching. These differences were reduced after matching, with an ASD > 0.10 only for sex, atrial fibrillation, intravenous thrombolysis and stroke etiology.

**Table 1:** Baseline characteristics of mild stroke patients according to treatment approach before and after propensity score-matching

	Before Propensity Score-matching			After Propensity Score-matching		
	MET group	Medical group	ASD	MET group	Medical group	ASD
Number	79	98	–	40	40	–
Age (years), mean ± SD	61.2 ± 14.4	65.9 ± 10.7	0.323	65.1 ± 11.6	65.0 ± 10.5	0.005
Sex (male)	52 (65.8)	72 (73.5)	0.160	27 (67.5)	29 (72.5)	0.105
Vascular risk factors						
Atrial fibrillation	21 (26.6)	13 (13.3)	0.300	9 (22.5)	7 (17.5)	0.112
Hypertension	51 (64.6)	62 (63.3)	0.027	28 (70.0)	28 (70.0)	NA
Hyperlipidemia	10 (12.7)	19 (19.4)	0.201	6 (15.0)	5 (12.5)	0.075
Diabetes	11 (13.9)	31 (31.6)	0.508	10 (25.0)	11 (27.5)	0.072
Smoking	17 (21.5)	34 (34.7)	0.319	8 (20.0)	9 (22.5)	0.006
Basement measurements						
Systolic blood pressure, mean ± SD	139.4 ± 20.9	147.0 ± 23.8	0.361	144.7 ± 23.7	143.4 ± 21.5	0.006
Admission NIHSS, median (IQR)	7 (5–8)	4 (2–6)	0.868	5.5 (4–7)	5.5 (4–7)	NA
ASPECTS, median (IQR)	9 (8–10)^a^	9 (8–10)	0.098	9 (8–10)	10 (8.25–10)	0.033
Good collateral circulation^b^	54 (68.4)	70 (71.4)	0.066	27 (67.5)	26 (65.0)	0.052
Onset to imaging (min), median (IQR)	300 (215–390)	300 (180–480)	0.432	284 (225–386)	240 (180–360)	0.057
Onset to puncture (min), mean (range)	314 (10–995)	NA	NA	284 (10–758)	NA	NA
Puncture to reperfusion (min),median (IQR)	75 (54–95)	NA	NA	75 (55–93)	NA	NA
Intravenous thrombolysis	23 (29.1)	11 (11.2)	0.391	13 (32.5)	10 (25.0)	0.164
Etiology						
Large artery atherosclerosis	41 (51.9)	85 (86.7)	0.693	29 (72.5)	31 (77.5)	0.099
Embolism	29 (36.7)	11 (11.2)	0.525	9 (22.5)	7 (17.5)	0.103
Others/undetermined	9 (11.4)^c^	2 (2.0)	0.292	2 (5.0)	2 (5.0)	NA
Site of occlusion						
ICA	5 (6.3)	25 (25.5)	0.400	4 (10.0)	5 (12.5)	0.069
MCA M1	49 (62.0)	39 (39.8)	0.455	21 (52.5)	21 (52.5)	NA
MCA M2	12 (15.2)	14 (14.3)	0.025	7 (17.5)	6 (15.0)	0.069
Tandem	13 (16.5)	20 (20.4)	0.106	8 (20.0)	8 (20.0)	NA

Value were showed as n (%) if not mentioned

*ASD* absolute standardized difference, *IQR* interquartile range, *SD* standard deviation, *NA* not applicable

^a^Two missing value were replaced by median; ^c^dissection (2 cases), thrombophilia (2 cases), cryptogenic stroke (5 cases)

^b^MET group was assessed according to digital subtraction angiogram, while medical group according to MR/CT resource angiogram

### Clinical outcomes

**Fig. 1:**
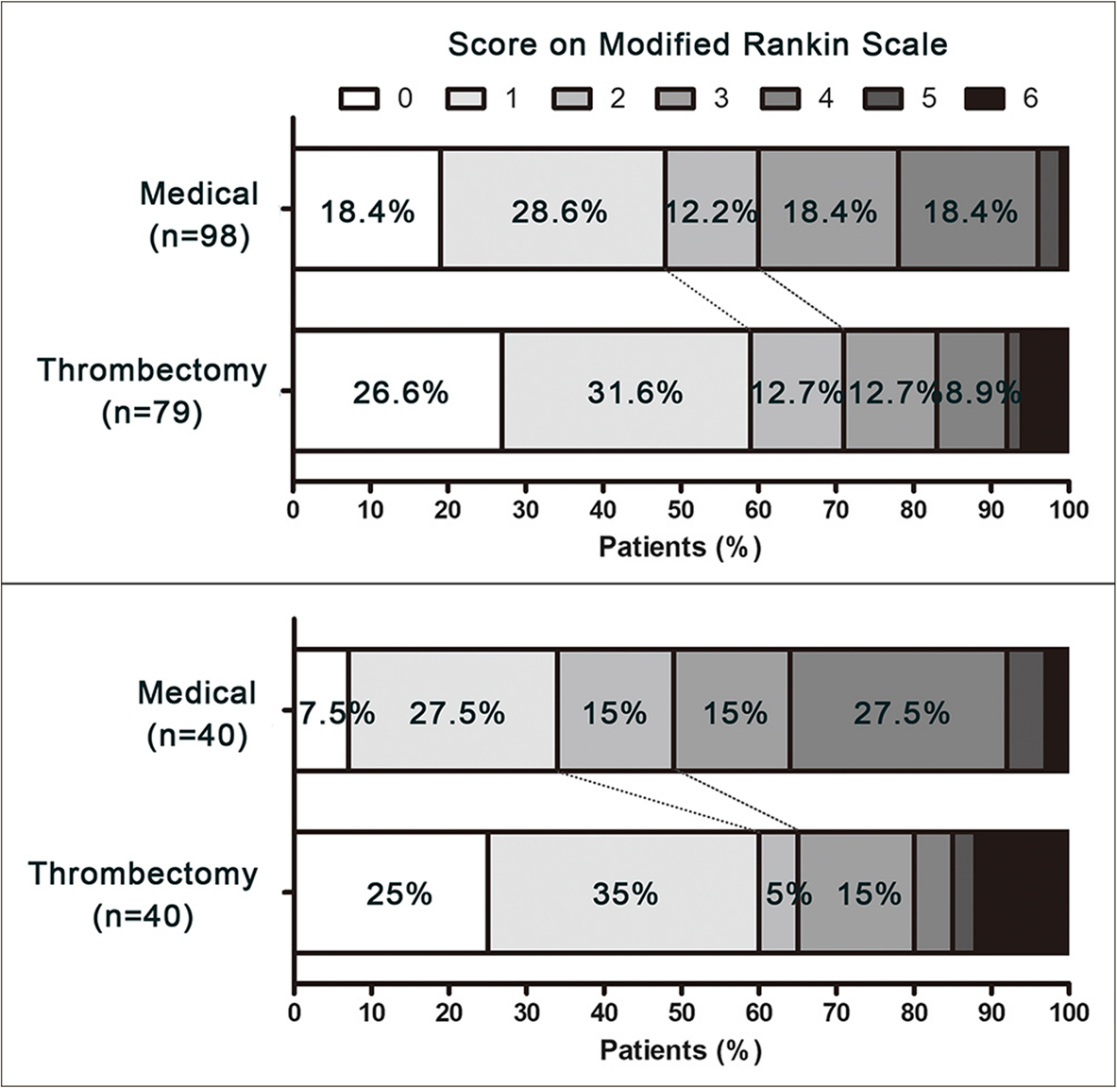
mRS at 3 months follow-up of stroke patients treated by thrombectomy and medical approach

There was no difference in crude comparison of primary outcome (mRS 0–1) between MET and medical group (58.2% versus 46.9%; *P* = 0.13); of the secondary outcome, MET group had more substantial sICH risks (10.1% versus 2.0%; OR, 95% confidence interval [CI]: 5.4, [1.11, 26.24]; *P* = 0.02), but favorable outcome (mRS 0–2: 70.9% versus 59.2%; *P* = 0.10) and mortality at 3 months (5.1% versus 1.0%; *P* = 0.17) did not show substantial difference. However, multivariable logistic regression analyses showed a statistically significant association of MET with good outcome (OR, 95% CI: 3.23, [1.35, 7.73]; *P* = 0.008) and favorable outcome (OR, 95% CI: 2.59, [1.06, 6.33]; *P* = 0.03), and not with a higher sICH risk (OR, 95%CI: 3.05, [0.44, 21.23]; *P* = 0.25). After the PS matching, the effect of MET on good outcome remained significant (OR, 95%CI: 2.78, [1.12, 6.89]; *P* = 0.02), and additional adjustment for variables with ASD > 0.10 did not affect the statistical significance of this association (OR, 95%CI: 3.20, [1.22, 8.37]; *P* = 0.01). (See Table 2 and Fig. 1).

**Table 2:** Odds ratios for clinical outcome by mechanical thrombectomy compared to medical therapy

	mRS 0–1 at 3 months			mRS 0–2 at 3 months			sICH at 48 h			Mortality at 3 months		
	OR	95% CI	*P* value	OR	95% CI	*P* value	OR	95% CI	*P* value	OR	95% CI	*P* value
Crude analysis	1.57	0.86 to 2.86	0.13	1.67	0.89 to 3.15	0.10	5.40	1.11 to 26.24	0.02	5.17	0.56 to 47.25	0.17
Multivariable analysis^a^	3.23	1.35 to 7.73	0.008	2.59	1.06 to 6.33	0.03	3.05	0.44 to 21.23	0.25	2.19	0.13 to 37.08	0.58
PS matching^b^	2.78	1.12 to 6.89	0.02	1.85	0.75 to 4.55	0.17	2.71	0.49 to 14.90	0.43	4.33	0.46 to 40.60	0.35
PS matching with additional adjustments^c^	3.20	1.22 to 8.37	0.01	2.08	0.79 to 5.47	0.13	3.08	0.45 to 20.69	0.24	4.85	0.48 to 49.04	0.18

*CI* confidence interval, *OR* Odd Ratio, *PS* propensity score

^a^Adjusted for age, sex, medical histories (atrial fibrillation, hyperlipidemia, diabetes), smoking, systolic blood pressure, admission NIHSS, time of onset to imaging, intravenous thrombolysis, stroke etiology and site of occlusion

^b^PS-matched sample included 40 pairs with 1:1 ratio

^c^Adjusted for Sex, atrial fibrillation, intravenous thrombolysis and stroke etiology in mRS outcome and mortality; adjusted for age, sex, atrial fibrillation, diabetes, intravenous thrombolysis, ASPECTS, time of onset to imaging and stroke etiology in sICH

### Meta-analysis

Of the 2484 records retrieved through database screening and other resources, 5 studies (this study named ‘Shang 2018’) were included in the final analysis (see in Additional file 1: Figure S1). [8, 10, 11, 17, 18] A total of 733 cases were included in the studies, 226 of which were comprised of 113 matched pairs. The analysis was conducted according to the NIHSS cutoff 8 or 5, and to compare endovascular treatment and medical therapy irrespective of rt-PA. Two studies were included in the comparison of MET versus medical treated patients with NIHSS ≤8 and LVO. When compared to patients who did not receive endovascular recanalization, patients treated with medical therapy had the similar functional outcome and procedural complications. (see Additional file 1: Figures S2 and S3). Four studies compared the clinical outcomes of patients with NIHSS ≤5 subgroup, the meta-analysis failed to find a correlation between endovascular treatment and clinical outcomes (see Additional file 1: Figures S4 and S5). All tests for subgroup difference did not find substantial heterogeneity.

## Discussion

In the present study, we found that endovascular thrombectomy was effective for improving good functional outcome, although it appeared to increase the incidence of symptomatic intracerebral bleeding, for stroke patients with mild deficits and proximal anterior LVO.

Mild stroke patients with LVO showed a worse prognosis and higher mortality compared with stroke patients without evidence of LVO. [19, 20] Thus, it was suggested that mild stroke patients presenting with proximal arterial occlusion should not be considered as MIS. [21] In a study of 204 eligible mild stroke patients with proximal LVO who did not receive any recanalization approaches, Mokin et al. reported that only 62% of patients were able to ambulate independently (mRS 0–3) at discharge. [22] By contrast, in the present study of a similar cohort of patients treated with endovascular thrombectomy, 70.9% exhibited functional independence (mRS 0–2) at 3 months, 10.1% showed sICH, and 5.1% all-cause mortality. In support, in a subset of stroke patients (NIHSS < 8) treated with MET, Dargazanli et al. reported 78.3% for 3-month favorable outcome and 5.1% for mortality. [23] Furthermore, in MET treated patients with an NIHSS ≤8, Pfaff et al. reported 63.6% for favorable outcome, 6.1% for sICH, and 9.1% for death. [24] Thus, overall these studies support that endovascular treatment for MIS and proximal LVO is favorably effective, as observed for intravenous thrombolysis. [25]

However, the proven evidence for the efficacy of thrombectomy therapy in MIS is still not available, and there is considerable heterogeneity among studies. For example, a pooled analysis from five recent randomized trials reported a negative finding in patients with an NIHSS ≤10 and LVO for thrombectomy compared with medical treatment. [26] Similarly, in a recent, larger multicenter intention-to-treat cohort study in MIS patients with an NIHSS < 8, there was no consistent improvement in good and favorable outcome. [8] As a result, further randomized controlled trials are needed to solve the uncertainty.

The main limitation of our study was the non-randomized design, and the limited number of patients may have underpowered our interpretation. Also due to the small sample size in this study, we did not further present the thrombectomy prognosis of patients with NIHSS ≤5, which was widely recognized as mild ischemic stroke, although the primary outcome was still positive.

As a highlight of this study, we focused on the meta-analysis of the efficacy and safety of endovascular treatment for mild stroke, and found no substantial differences of endovascular versus medical treatment for patients with NIHSS 8 or 5 on matching or not. The specific reasons of inconsistence with our data are as follows: first of all, all studies diagnostic criteria were not completely consistent, e.g. some studies recruited cases with the posterior circulation infarction, and it is well known that these patients might progress rapidly and had poorer outcome compared with those in anterior circulation, which could have an effect on the outcomes. Also, the definition criterion of symptomatic bleeding was variable, and there were different endovascular procedures between studies, including intra-arterial thrombolysis, angioplasty and mostly thrombectomy, which might confuse the practical effects of thrombectomy. Therefore, it is recommended to obtain more detailed information of other studies and stratify the meta-analysis, which may be accessible to original nature.

## Conclusions

Our data have found a statistically significant benefit of endovascular thrombectomy for MIS patients with proximal LVO in anterior circulation, but it should be confirmed by further high quality trials.

## Additional files

Additional file 1: **Figure S1.** PRISMA (Preferred Reporting Items for Systematic Reviews and Meta-Analyses) flow-chart of search strategy. **Figure S2.** Forest plot of outcomes between endovascular treatment and medical therapy in patients with NIHSS ≤8. **Figure S3.** Forest plot of outcomes between endovascular treatment and medical therapy in matched patients with NIHSS ≤8. **Figure S4.** Forest plot of outcomes between endovascular treatment and medical therapy in patients with NIHSS ≤5. **Figure S5.** Forest plot of outcomes between endovascular treatment and medical therapy in matched patients with NIHSS ≤5. (DOCX 2 kb)

## Data Availability

The data is available on request to the corresponding author.

## Abbreviations

ASPECTS: Alberta stroke program early CT score
CI: Confidence interval
CTA: computed tomography angiography
ICA: Intracranial carotid artery
LVO: Large vessel occlusion
MCA: Middle cerebral artery
MIS: Mild ischemic stroke
MRA: Magnetic resonance angiography
mRS: modified Rankin Scale
NIHSS: National institute of health stroke scale
OR: Odds ratio
RCT: Randomized control trials
sICH: symptomatic intracranial hemorrhage
