# Supplementary material for: Efficacy and safety of endovascular thrombectomy in mild ischemic stroke: results from a retrospective study and meta-analysis of previous trials
Source: BMC Neurol. 2019 Jul 5;19:150. doi: 10.1186/s12883-019-1372-9 (PMC6610891; doi:10.1186/s12883-019-1372-9)
Supplement: Supplementary file 1 — Figure S1. PRISMA (Preferred Reporting Items for Systematic Reviews and Meta-Analyses) flow-chart of search strategy. Figure S2. Forest plot of outcomes between endovascular treatment and medical therapy in patients with NIHSS ≤8. Figure S3. Forest plot of outcomes between endovascular treatment and medical therapy in matched patients with NIHSS ≤8. Figure S4. Forest plot of outcomes between endovascular treatment and medical therapy in patients with NIHSS ≤5. Figure S5. Forest plot of outcomes between endovascular treatment and medical therapy in matched patients with NIHSS ≤5. (DOCX 2 kb) [file 12883_2019_1372_MOESM1_ESM.docx]

**Fig.1** PRISMA (Preferred Reporting Items for Systematic Reviews and Meta-Analyses) flow-chart of search strategy

Article excluded after full-text screen (n=23):

From the same center (n=1)

Case report (n=5)

No endovascular therapy (n=12)

Not NIHSS ≤8 (n=5)

Records identified through database searching (n=2482)

Additional records identified through other sources (n=2)

Records after duplicates eliminated (n=2484)

Records screened

(n=2484)

Records excluded

(n=2456)

Full-text articles assessed for eligibility (n=28)

Studies included in final meta-analysis (n=5)

**Fig. 2** Forest plot of outcomes between endovascular treatment and medical therapy in patients with NIHSS ≤8





**Fig.3** Forest plot of outcomes between endovascular treatment and medical therapy in matched patients with NIHSS ≤8





**Fig.4** Forest plot of outcomes between endovascular treatment and medical therapy in patients with NIHSS ≤5





**Fig.5** Forest plot of outcomes between endovascular treatment and medical therapy in matched patients with NIHSS ≤5
